# In critique of anthropocentrism: a more-than-human ethical framework for antimicrobial resistance

**DOI:** 10.1136/medhum-2021-012309

**Published:** 2022-03-23

**Authors:** Jose A Cañada, Salla Sariola, Andrea Butcher

**Affiliations:** 1 Sociology, Philosophy and Anthropology, University of Exeter, Exeter, UK; 2 Sociology, University of Helsinki, Helsinki, Uusimaa, Finland

**Keywords:** medical ethics/bioethics, medical humanities, microbiology, public health, social science

## Abstract

Antimicrobial resistance (AMR) is often framed as a One Health issue, premised on the interdependence between human, animal and environmental health. Despite this framing, the focus across policymaking, implementation and the ethics of AMR remains anthropocentric in practice, with human health taking priority over the health of non-human animals and the environment, both of which mostly appear as secondary elements to be adjusted to minimise impact on human populations. This perpetuates cross-sectoral asymmetries whereby human health institutions have access to bigger budgets and technical support, limiting the ability of agricultural, animal health or environmental institutions to effectively implement policy initiatives. In this article, we review these asymmetries from an ethical perspective. Through a review and analysis of contemporary literature on the ethics of AMR, we demonstrate how the ethical challenges and tensions raised still emerge from an anthropocentric framing, and argue that such literature fails to address the problematic health hierarchies that underlie policies and ethics of AMR. As a consequence, they fail to provide the necessary tools to ethically evaluate the more-than-human challenges that the long list of actors involved in managing AMR face in their everyday practices. In response to such shortcomings, and to make sense of these challenges and tensions, this article develops an ethical framework based on relationality, care ethics and ambivalence that attends to the more-than-human character of AMR. We formulate this approach without overlooking everyday challenges of implementation by putting the framework in conversation with concrete situations from precarious settings in West Africa. This article concludes by arguing that a useful AMR ethics framework needs to consider and take seriously non-human others as an integral part of both health and disease in any given ecology.

## Introduction

Historically, human health, veterinary health and environmental health have been addressed by independently formulated disciplines, such as medicine, public health, veterinary science or environmental sciences. Such division has been visible within research, policy and practice alike (Zinsstag *et al* 2012). Between them, the preservation of animal and environmental health has been given less priority than human health and only considered relevant to the extent that they impact the health of humans. The last decade has seen a shift in these dynamics—at least conceptually and, to a certain extent, also organisationally—with the popularisation of One Health, a multidisciplinary approach that has the inseparability of the health of humans, non-human animals and the environment as its core premise.

The conceptualisation of One Health dates back to the 1960s, then termed ‘One Medicine’ (Cassidy 2017) and the first One Health initiatives were introduced in the early mid 2000s in connection to emerging zoonotic diseases with pandemic potential, like influenza (Chien 2013). However, it was the 2015 global initiative to tackle antimicrobial resistance (AMR) that provided One Health with a clearly defined policy space. Global AMR policy initiatives are best represented by the publication of the ‘Global action plan on antimicrobial resistance’ (World Health Organization 2015). This plan was developed by the so-called tripartite collaboration involving the WHO, the Food and Agriculture Organization and the World Organisation for Animal Health. Framing AMR as a One Health issue makes explicit the sharing of microbes between human and non-human animals, and the impact of antimicrobial pollution on microbial ecologies1 in the environment. As defined in AMR policy initiatives, the increased presence of resistant bacteria is associated with the extensive use of antibiotics in medicine, farming and agriculture, and to the uncontrolled pouring of antibiotic waste into the environment. This combination turns multisectoral involvement into one of the key objectives for AMR initiatives, requiring involvement from human and veterinary health, agriculture and livestock, and environmental sectors. The One Health approach also widens the scope of what can be considered drivers of AMR, with antibiotic use being part of wider complex ecologies that include environmental factors. One Health’s conceptual proposal is in many respects revolutionary, as it calls for a profound reorganisation of well-established disciplinary boundaries, with the consequences that this has for scientific and lay understandings of medicine and health on our planet.

Global health initiatives for AMR present an ambitious enterprise that requires radically altering some of the basic underpinnings of Western human and veterinary medicine, which heavily relies on antibiotic medicines to deal with a considerable amount of health problems in humans and non-human animals. Given the scale and potential inequal impact of these changes, AMR initiatives present an ethical element that has generated considerable academic discussion (Aiello, King, and Foxman 2006; Anomaly 2009; Krockow and Tarrant 2019; Littmann and Viens 2015; Marcus, Clarfield, and Moses 2001; Millar 2011; Parsonage *et al* 2017; Rollin 2001; Rump *et al* 2018). These discussions have almost exclusively focused on the impact of change in the use of antimicrobials on human health, largely ignoring the One Health framing of AMR. The following section expands on the shortcomings of this human-centred perspective in order to identify the necessary elements for an ethical framework of AMR that takes into account the basic premises of One Health.

## Anthropocentrism in One Health and AMR ethics

Despite One Health’s conceptual ambition, its actual implementation has not really challenged established hierarchies among humans, non-human animals and the environment, with One Health policy remaining predominantly anthropocentric. Anthropocentrism is a perspective that Thompson (2017, 78-79) has defined as ‘a view about the ultimate nature of reality that prioritises the existence of human beings’, with ethical anthropocentrism considering humans as ‘morally superior to everything else in the natural order’, attributing them a ‘a special and unique moral importance’. Anthropocentrism in AMR is especially visible in global policy initiatives. A study by Kamenshchikova *et al.* (2019) shows that, despite its embrace of the One Health approach, AMR policy has not really committed to its conceptual premises—the focus remains on improving, sustaining and promoting human health. In their analysis of AMR documents with a One Health approach they found non-human animals to be characterised as either ‘a resource for human health’ or ‘as potential carriers of disease’ (Kamenshchikova *et al*. 2019, 310). Furthermore, attention was mostly directed at domesticated animals involved in human economies, that is, agriculture and food production. In the documents they analysed, Kamenshchikova *et al* found that the environment remained either unaddressed, or was included as a generic reference lacking clear formulation or operationalisation of how the environment ought to be addressed. These references were therefore unable to provide a clear vision of what AMR policy interventions in environmental health as inseparable from human and non-human animal health might look like.

In their analysis, Kamenshchikova *et al* found reports of antibiotic use in clinical and agricultural settings to be anthropocentric, with causality established unidirectionally; humans were presented as sufferers of disease, while non-humans were defined as risk factors that contribute to that suffering. We use the work of Kamenshchikova *et al* as inspiration for addressing the ethical dimensions of AMR. In relation to practices of diagnosis, caring and treatment, our critique understands anthropocentrism in AMR ethics as those practices being conducted in a way that prioritises the safety of humanity while subjugating the health of non-humans and the environment to that safety. From an anthropocentric perspective, disregarding non-human actors in AMR ecologies might be wrong if it fails to solve the health threat posed to humans and their economies, but is never ethically problematic. We argue that an anthropocentric attitude *ought* to be considered as ethically problematic because it does not consider the ecological implications of putting humans first, instead subjugating non-human suffering to human safety, while at the same time failing to provide a framework that recognises the interdependent aspects of health that the One Health formulation of AMR is based on.

While ethical discussions in One Health have generally been the subject of anthropocentric critiques, with its multispecies premises having received considerable scrutiny (eg, Johnson and Degeling 2019; Rock and Degeling 2015; Herten, Bovenkerk, and Verweij 2019; Capps 2019; Hermesh, Rosenthal, and Davidovitch 2019; Nieuwland and Meijboom 2019), AMR ethical discussions (see Aiello, King, and Foxman 2006; Anomaly 2009; Krockow and Tarrant 2019; Littmann, Buyx, and Cars 2015; Marcus, Clarfield, and Moses 2001; Millar 2011; Parsonage *et al* 2017; Rollin 2001; Rump *et al* 2018) have not been specifically exposed to such critiques. Instead, they have predominantly retained an anthropocentric outlook, with animals appearing as economic actors in the food production chain whose lives are less valuable than those of humans and with the environment being largely absent and lacking operationalisation, in a similar vein to the findings of Kamenshchikova *et al*. (2019) introduced above regarding policy. Awareness of this anthropocentric bias is not completely absent though; for example, Littmann and Viens (2015) state that most ethical accounts of AMR ignore the role played by agriculture and farming, as well as the need to engage with animal and environmental ethics. However, this recognition does not really lead to any attempt at a corrective action. Littman’s and Viens’ paper—the most highly cited paper on AMR ethics at the time of writing—is critical of how AMR’s impact on farming and agriculture is given secondary priority and yet it reproduces the same shortcoming that they diagnose by focusing recommendations and conclusions exclusively on human health. Furthermore, their concern with animal health remains attached to farming and food-producing activities, rather than with how animals and the environment are affected by AMR, thus retaining an anthropocentric perspective.

Anthropocentrism is also problematic as it is an intrinsic driver of the AMR crisis. The broader idea of human primacy over other living beings and the environment, and the assumption of humanity’s right to longer and healthier lives (Broom *et al* 2020), no matter the cost for other animals has greatly contributed to the massive use of antibiotics. Such exceptionalism is related to what Marques (2020), 391) has called an ‘anthropocentric illusion’ that postulates progress and development as the only possible future for humanity and that, from an ecological perspective, presumes ‘that man generally adapts his *habitat* to his ends, unlike other species which, generally, adapt to him’ (Marques 2020, 393), an idea of which AMR is one of its most serious examples (Marques 2020). Here, we do not argue against the rights of human individuals to live healthily but problematise how such rights are embedded in notions of human exceptionalism that do harm to other animals and the environment, and eventually to humans themselves. Furthermore, going beyond human health is not only a conceptual matter, but also a key strategic element with the objective of achieving multisectoral involvement in AMR initiatives. For example, communication and awareness campaigns have thus far focused disproportionately on individual prescription for humans and patient use, but campaigns that go beyond human health have the potential to involve a much wider sector of society in addressing the issue of AMR (Thornber and Pitchforth 2021).

Nieuwland and Meijboom (2019) have tried to challenge this exceptionalism by opposing One Health with *One Welfare*. They ask, is it enough to recognise the interconnectedness of non-human health for ensuring human health or do we need to go beyond human health and interests to ensure non-human welfare? We think that a tentative answer to that question can be found in the work of Johnson and Degeling (2019), which suggests that for One Health to live up to its potential, it is necessary to go beyond health2 as an interconnection between humans, non-human animals and the environment. Along the same lines, we would like to add—as we argue throughout the paper—that this requires a shift in the unit of analysis from the individual or the species towards the ecology as an element that includes both human and non-human elements. Moving in that direction requires policy to recognise the multispecies character of AMR ecologies, and to consider ethics beyond the impact of AMR on humans by taking into consideration non-human agency. This also requires considering microbes as central actors, whether they have a pathogenic, beneficial or mutualist character. Microbes should be defined in relation to the biotic role they play in the health of other elements within a given ecosystem that determines the health or disease status of the different human and non-human actors involved. We argue that the failure to consider AMR ethics from an ecological perspective stems from a lack of commitment to the most basic premises of One Health.

In the next section, the paper proceeds by introducing a non-anthropocentric framework for AMR ethics. This framework is based on three independent but complementary approaches coming from feminist literature in Science and Technology Studies: relationality, ambivalence and care ethics. We then dedicate a section to each of those three elements in which we develop their conceptual contribution while bringing them into conversation in a tiered manner. In the first of those three sections, we expand on the need for a relational approach and its role in understanding how value of different human and non-human actors emerges as part of AMR ecologies. In the second, we present how such shifts in value play an important role in caring for non-human others. This helps to illustrate how practices of care in multispecies AMR work in locally situated settings where actual intervention is constrained by precarity, lack of resources and economic needs. Third, we discuss ambivalence as a way of understanding the affective relations between humans and non-humans as a result of displacing the human from its superior moral standing. Throughout these three sections, we interweave our critique of anthropocentrism with the theoretical formulation of our ethical framework for AMR, assisted by the use of empirical examples from our fieldwork, where we followed the implementation of global AMR policies in West Africa. We conclude the paper by arguing that a useful AMR ethics framework needs to consider and take seriously non-human others as an integral part of both health and disease in any given ecology.

## A non-anthropocentric framework for AMR

In the previous section, we have built on One Health critiques to review AMR ethical discussions and concluded that they are insufficient for making sense of the complex ecologies that emerge in relation to AMR. To address this insufficiency, we draw on existing multispecies ethics in feminist Science and Technology Studies to formulate a non-anthropocentric ethical framework that assists in evaluating the complex situations that emerge in the context of AMR. Our reading of AMR in this article is non-anthropocentric in the sense that it problematises the prioritisation of human health over other important non-human actors present in AMR ecologies. However, we do not advocate a flat understanding where the healths of human and non-human animals, microbes, and the environment have equivalent value; rather we advocate for an approach to decentring human exceptionalism in AMR ethics that relationally engages with the situated conditions that give way to different hierarchies inside AMR ecologies. This way of understanding anthropocentrism in relation to AMR exposes challenges in regards to more-than-human3 forms of inequality—or perhaps a lack of more-than-human solidarity, using the words of Rock and Degeling (2015)—that emerge in specific settings where AMR management involves antibiotic treatment.4 In this paper, we argue that engaging in antibiotic treatment while reconceptualising the relations that bind the health of human, non-human animals, and the environment requires ethicopolitical commitments to address those inequalities for which we lack a fitting framework.

The framework we propose consists of three differentiated but complementary ethical approaches that address three specific issues that emerge as a result of our anthropocentric critique: the need for ecological thinking, the need to articulate non-anthropocentric attitudes in close engagement with non-humans and the potential flatness emerging from debunking human exceptionalist hierarchies. To address the first one, we propose a relational approach to ethics that highlights the situatedness of AMR ecologies, as formulated in the work of Whatmore (1997). Relationality helps us to resituate the different human and non-human elements involved in AMR after placing the ecology as the central unit of analysis. Committing to an ecological notion for an AMR ethic means having to evaluate the value of each actor involved in relation to the rest of the elements present in an ecology, including humans and non-humans. Second, we rely on a care ethics approach with a more-than-human sensitivity that attends to the multispecies character of AMR, as formulated by Puig de la Bellacasa (2017). A care ethics approach provides a rationale for the direct engagement between humans and non-humans that emerges in more-than-human ecologies by giving specific value to experiences of caring for others as valid and valuable forms of moral decision-making, often entailing negative and positive ways of relating to each other simultaneously. Finally, to make sense of those potentially ambivalent affects and in order to avoid the flat understanding of value that can result from questioning the fixed hierarchies associated to anthropocentrism, we rely on Haraway's (2008) *ethics of killing*, which bring a regardful attitude towards multispecies encounters that entail a range of affective interactions. This involves paying attention to how ambivalent affective relations do not need to create fixed hierarchies where some lives are always superior to others or where all lives are regarded as having the same value.

We develop this framework vis-à-vis examples from the field to illustrate why the predominant anthropocentric perspective in AMR ethics requires revision. Our examples show how relevant AMR actors deal with multispecies encounters in the field while considering evolving recommendations and knowledge about AMR. It is worth clarifying that the actors featured in our examples do not explicitly or consciously defend non-anthropocentric forms of understanding AMR themselves. Indeed, in the same vein as policy initiatives and existing AMR ethics discussions, AMR management remains mostly anthropocentric in its practices, including those undertaken by the actors in our examples. What the examples do illustrate is how an anthropocentric AMR ethic would find little that is intrinsically wrong with how AMR was managed in those situations. At most, the problem would be framed as a technical one of not being able to control or get rid of disease. We argue that considering non-human health as secondary is unethical both for humans and non-humans since it prevents the creation of spaces where resistance can be managed in all its complexity, while continuing to disregard the relevance of non-human actors and relegating them to a mere secondary role. By analysing the examples using our suggested framework, we try to demonstrate that a more-than-human relational perspective is not only of value for decision-makers to address AMR more ecologically, but also necessary to recognise that humans are part of wider ecologies on the same terms as non-human others, something that demands the revision of existing multispecies hierarchies.

Empirically, we focus on precarious settings where changes in antibiotic use promoted by global initiatives present a heightened challenge given the role antibiotic practices have as compensating elements for the lack of basic infrastructures for humans, non-human animals and the environment (Denyer Willis and Chandler 2019). To illustrate our proposed framework, we use material from ethnographic fieldwork conducted between February 2019 and March 2020 in the West African countries of Benin and Burkina Faso, two countries that have struggled to develop and implement a national AMR action plan, and where the balance of funding between the three spheres of One Health is heavily weighted towards human health. Our material includes the analysis of interviews with scientists, policymakers and healthcare staff; focus groups with veterinarians and breeders; ethnographic diaries from fieldwork; and international and national policy documents. During our fieldwork, we spent time following scientists involved in AMR knowledge production, international projects and initiatives, and policymaking. In addition, we conducted observation on three livestock agribusinesses ranging from 5-day visits on farms producing poultry, guinea fowl, swine, cattle, and small ruminants in Burkina Faso, to a 3-week stay on a Beninese layer poultry farm. Finally, we also conducted nine focus groups with vets and breeders. The examples we use are based on a combination of our own observations and the stories told to us by our informants, who experienced them first-hand. The study on which this article draws did not include patient and public involvement.

## Relationality and value in more-than-human ecologies

The first element of our framework is relationality, which helps in understanding what happens to the interconnections between humans, non-human animals and the environment when we look at them through an ecological lens. Relational ethics is a line of thought to which the work of Whatmore (1997) is especially worth mentioning. For Whatmore, humanist (anthropocentric in our vocabulary) and masculinist ethics have historically excluded feminist and environmentalist ethics. In the context of AMR, we argue that an anthropocentric outlook perpetuates the humanist and masculinist values critiqued by Whatmore. Excluding feminist and environmentalist ethics—both central elements for our framework—works against the objective of developing a more ecologically minded understanding of AMR. Attending to feminist and environmentalist ethics means extending the body politic beyond the human subject and placing emphasis in practised and embodied processes. This displaces the fixed and bounded contours of the ethical community, moving away from individualist understandings of responsibility, something that has been identified as an issue in global initiatives of AMR (Chandler 2019). Whatmore’s relational approach advocates for an understanding of community that is necessarily hybrid and is ‘conceived as occupying narrow lines of force that allow us to pass with continuity from the local to the global, from the human to the nonhuman, through partial and unstable orderings of numerous practices, instruments, documents, and bodies’ (Whatmore 1997, 47). This hybrid understanding disrupts pure understandings of culture and nature as separate ontological zones, breaking also with anthropocentric framings and human exceptionalism. Therefore, committing to a relational understanding of ethics in relation to AMR ecologies means not only acknowledging interconnections between humans, non-human animals and the environment, but understanding the way the value of the different elements in those three interconnected areas emerge as a result of their being together in the same ecology.

To illustrate how value emerges relationally between more-than-human actors in a given ecology, we turn to an example narrated by a veterinarian and former director of a Beninese public laboratory for animal health. In the example, our informant told us about his experience participating in the struggle to control the impact of a resistant parasite, the *Boophilus microplus*, which arrived in Benin with the importation of Girolando cows, a breed created in and exported from Brazil. The *B. microplus* is a tick that transmits several diseases affecting cattle and has considerable economic, veterinary and medical impact worldwide (Mehlhorn 2016). Of those diseases, it is *Babesia bovis* that is most significant, a blood parasite that is transmitted to cattle through larval attachment of *B. microplus*. The ticks attach not only to cattle but also to shepherds and cattle dogs, requiring treatment and making evident the ecological dimensions of the issue. Thus, the tick and the parasite are able to affect many actors in the ecology, both human and non-human, but the value attributed to the health of each is shaped relationally, instead of being fixed *a priori*. The example makes clear the need to consider more-than-human ecologies—rather than specific species or individual beings—as the site for intervention.

In our informant’s example, following the arrival of the *B. microplus* in Benin in the early 2000s (Biguezoton *et al* 2016), the rate of blood infections in dairy cows increased considerably. To address the issue, a trial-and-error process began in search of a solution. The first strategy relied on an increased dose of acaricides (a type of pesticide), targeting the vector instead of the blood parasite. Synthetic acaricides are the most common tool to fight the *B. microplus* since they offer a relatively quick and cost-effective suppression of tick populations (Abbas et al. 2014). However, long-term use of acaricides on a given population contributes to the development of resistance among ticks (Raynal et al. 2013). Furthermore, breeders and animal health experts in Benin also became concerned that the high doses of acaricide required to eliminate the ticks were having potentially negative impacts on the health of cows. In an attempt to find a strategy with less negative impacts on livestock health, several antimicrobials were tested that targeted the parasite instead, until an effective one was eventually found. However, this antimicrobial had negative effects on the milk of the cows5 and so its use was eventually prohibited, demonstrating that more value is deposited in the products of the agricultural activities, despite the improvement in the condition of the cows. Another potential solution for eradicating the resistant parasite was to burn the grass fields inhabited by *B. microplus*. However, this idea was discarded because the measures would have had an impact beyond the destruction of the grass itself, affecting the quality of soils and the presence of water (Neary and Leonard 2020), a scarce resource in West Africa. The decision to abandon this strategy also shows concern for the environmental element of the more-than-human ecology of which *B. microplus* forms a part.

By foregrounding the relevance of non-human actors like soil, water, milk and cows, this case demonstrates the importance of developing frameworks that address AMR ecologically rather than simply focusing on reducing the use of antimicrobials, which can also have unintended negative consequences as seen with the above example. The use of different tools to manage the health and the death of the different ecological actors involved (the cows, the tick, the parasite, the soil, the grass, the shepherds, the cattle dogs) leads to a relational understanding of the impact of intervention, which shifts value across actors depending on the impact of a given treatment or strategy. AMR as a specific challenge presents an opportunity for a critical and unique broadening of who and what we consider to be relevant actors for the sustainability of environment relations but, most importantly, it is an opportunity to recognise that the role of those actors is not fixed and depends on the context in which they are situated. The example above shows a concern for soil integrity (Krzywoszynska and Marchesi 2020; O’Brien 2020) as a key element of viable agricultural ecologies, while at the same time forces us to reflect on the objectification dynamics humans exercise on non-humans (Coope 2021) and how these are justified on the basis of economic production, for example, prioritising the milk the cows produce over their physical health and well-being. Visible in this is the complex transfer of value between different forms of life, showing how an ethical account of AMR that does not formulate its more-than-human aspect in relational terms can easily become partial and insufficient.

## More-than-human care ethics in diagnosis and treatment

Ecological thinking sets the framing for understanding value as constantly shifting in relations that evidence the shared health of humans and non-humans. This sharing means that addressing the health of one requires attending to the health of the other, calling for spaces where direct engagement is possible. For that engagement to be formulated in practical terms, we rely on more-than-human care ethics, as featured prominently in the work of Puig de la Bellacasa (2017). Care ethics is a feminist approach that purports the idea that women’s experiences in caring for others are valid and valuable forms of moral decision-making (Vázquez Verdera 2010). Bringing in a feminist approach also helps to compensate for the dominating character of humanist and masculinist ethics that a relational approach addresses. Puig de la Bellacasa’s formulation of care ethics also binds relational approaches to an understanding of more-than-human interaction that does not take for granted the value of different life forms or, put another way, that understands the value of any life not as an intrinsic characteristic but something that emerges in caring relations between members of a given ecology. More specifically, Puig de la Bellacasa argues for the use of care ethics in the context of the more-than-human ecologies that feature in practices of permaculture, a strategy of land management that takes inspiration from flourishing natural ecosystems. This implies thinking of ecologies as containers of humans and non-human animals, plants, air and water (all elements with recognised prominence in AMR dynamics), together with microbes as central actors in these assemblages, both as pathogens and as contributors to healthy microbiomes—as already argued above. For Puig de la Bellacasa (2017, 145), these more-than-human assemblages form webs ‘of living co-vulnerabilities’. Thinking in terms of shared vulnerability helps to counter notions of human superiority by considering how humans and non-humans support and depend on each other for healthy living, while also playing a role in any decline of that shared health. Being part of these health ecologies requires different actors to care for one another’s flourishing. We are aware that caring remains a human conceptualisation of certain practices, but it highlights the relational, affective and ambivalent aspects that brings human and non-human elements together as part of shared ecologies.

Care ethics also provides an approach in which first-hand experience is key in care provision, something that we argue is central to deciding treatment in precarious settings, both for humans and non-humans. Care involves a level of reciprocity that Puig de la Bellacasa illustrates with the example—that on some occasions also works as an analogy—of touch, something that instantly evokes a need for direct engagement with the object of care. The touch analogy can be made more concrete by the four types of encounters proposed by Tschakert (2020) to further more-than-human solidarity, and that represent an increasing coming into contact with non-human others: visual, corporeal, ethical and political encounters. Tschakert’s conceptualisation forces us to recognise the concrete need to take seriously our encounters with non-humans—including microbes—and reformulate politics as something that goes beyond interaction between humans as a way of unidirectionally managing the existence of non-human animal and environmental actors. Puig de la Bellacasa’s touch as analogy serves to summarise Tschakert’s four encounters in one: the visual recognition that does not objectify is combined with corporeal embodied encounters that in their enactment make evident an ethicopolitical mode of being in this world. Touch can serve as an alternative framework for intervention in the absence of diagnostic capacity evident in both our fieldwork sites (Cañada 2021).

For example, when diagnosing infections in poultry, the preferred option and global recommendation is for laboratory analysis to be performed alongside empirical observation of poultry litter6 and faecal matter (Buller *et al* 2020). While clinical diagnosis of bacterial disease in chickens is much less accurate than laboratory diagnosis (Hasan *et al* 2012), this type of specificity is generally unavailable to breeders in the studied countries who have limited access to laboratories and who often lack the financial resources for clinical diagnosis with veterinary consultants (Cañada 2021). Instead, farmers make their own diagnosis and treatment decisions, either according to their own knowledge and experiences of dealing with previous production diseases, or after consulting with peers (Butcher, Cañada, and Sariola 2021). In the treatment of poultry diseases, diagnosis is often made through observation of the poultry litter and faecal matter, and subsequent treatment usually addresses the entire flock as a single unit, making evident the ecological level of treatment. There are valid reasons for adopting this approach: if observation of litter and faecal matter determines the presence of a bacterial infection, there is a high probability that all birds have been exposed and are potentially infected— a consequence of the crowded cages and small spaces in which non-human animals bred for food are confined. For example, on the poultry layer farm where we spent time doing observations, measures were in place for isolating individual birds showing physical signs of disease or distress, something only noticeable if one is attentive to the physical and behavioural aspects of the birds. Hens displaying signs of fatigue, unable to feed or walk properly, or presenting with eye or skin infections, were removed from the main enclosure, housed in a small isolation cage with other sick birds, and treated with vitamins and (if deemed necessary) antibiotics. They would be returned to the main enclosure only when their symptoms had improved or when drug withdrawal was complete. However, bacterial infections such as salmonella or infectious bronchitis were diagnosed according to the colour and consistency of faecal matter in the litter, thus requiring the flock to be treated as a single unit. All these observations and treatment interventions are examples of close engagement with non-human others that are necessary given the absence of diagnostic capacity. In the absence of reliable infrastructures (Denyer Willis and Chandler 2019), touch as analogy for caring multispecies encounters serves as an alternative framework to inform and support more sustainable and realistic solutions—in comparison to options like laboratory diagnosis, which require heavy infrastructural investment—that do not put such a strain on precarious settings inhabited by fragile health systems, vulnerable patients and fragile survival economies.

Caring for non-human others does not exclusively consist of pleasant companionship, as is often the case with how human-companion or pet relations are framed. Puig de la Bellacasa (2017, 147) explains that caring ‘is not reducible to ‘feel good’ or ‘nice feelings’; repulsion is not incompatible with affectionate care’ or with utilitarian approaches to our relations with non-humans. Indeed, our informants talked ambivalently about regard and love for non-humans constantly in animal production settings. We found such expressions brought in a mix of emotions and attachment that can be interpreted as caring for economic production as well as for the affective relations established between a farmer, their livestock and the land. The owner of a farm of laying hens would talk dearly about the first tree he encountered and under which he and his family slept when they acquired the land where now a rudimentarily biosecure farm is situated, and where 20 000 chickens produce 12 000 eggs daily. His affectionate description of the area, the chickens and the buildings revealed a care relation that was both emotional but also economic: through its productivity it provided a higher living standard for him and his family, simultaneously increasing social status. Or for example a technician on the same farm who told us how he had wanted to breed chickens since he was a child due to the love he professed for them, but who still engaged in and supported non-caring strategies to keep the chickens passive to human control, (ie, by removing their beaks at birth or keeping them in crowded cages), making their exploitation possible through the disruption of their agency, something that Beldo (2017) has called metabolic labour. On another occasion, a technician on the same farm inspecting a chicken for parasites fondly stroked the afflicted bird and spoke of his love for the chickens in his care, while simultaneously asserting sickness and discomfort to be an evitable part of the conditions of intensive production to which they were subjected. An ethical framing of AMR requires considering the complexities of such engagements between humans and non-humans, which require relational evaluation and a close understanding of the histories and encounters that make health a shared element.

## Ambivalence in regardful multispecies relations

We make sense of that mix of care, exploitation, and positive and negative affects that populate more-than-human relations in AMR by relying on the work of Haraway (2008), who has proposed an *ethics of killing* that does not reject, exclude or denounce death. Rather, it rejects the idea that some creatures are killable by virtue of the circumstances into which they are birthed. In more-than-human contexts like AMR, it is common to see that certain lives are valued more than others by virtue of those circumstances, as was clear from the example above in which the health and well-being of cows came second to the commercial value of their products. According to Haraway’s ethics of killing, the objective is to kill well, which requires attending to ‘the ethical injunctions to be curious and to hold in mutual regard’ (Ginn 2014, 538). This is therefore not just a question of rights intrinsic to the animals and their existence in the sense formulated by early animal rights activists (Singer 1990) but a question of articulating human–non-human relations by recognising their complexity and interdependence. For Haraway (2008, 53), it is a question of how ‘may a human enter into a rights relationship with an animal’ without prefixing what that relation is and considering that any demands more-than-human partners make of each other can be life-changing for all partners.

Ambivalence helps here to make sense of the flatness that can result from breaking with established hierarchies and creating spaces where the value attached to certain species or environmental elements is no longer fixed and bounded. Relational ambivalence is not only ethically important, but also useful and necessary to retain an awareness of difference between distinct types of health and forms of life. Addressing these differences requires retaining the ecology as the central unit of analysis, something that contrasts with existing discussions about AMR ethics, which often take for granted the hierarchies of worth that place humans as the absolute priority in the implementation of AMR policy. Our framework calls for a non-fixed, non-flat understanding of the way the health of humans, non-human animals and the environment are prioritised over one another. Thus, our framework serves as a call to know, study, recognise and give regardful attention to how hierarchies in AMR are the result of direct interaction between humans and non-humans. Along these lines, rather than supporting the elimination of AMR, or perpetuating the reductionist understanding of microbes as pathogens to eliminate, the framework we present attempts to formulate ways of coexisting with microbes that are potentially resistant to antimicrobials—particularly where their presence supports the health of an ecology—in ethical and sustainable ways that take seriously the basic premises of One Health.

This allows for an ambivalent understanding of multispecies difference that is useful in evaluating antibiotic action, the exploitation of animals for food production, and the discharge of different types of waste into the environment, as part of ethical and regardful more-than-human interactions. This is the case with the growing focus on the development of probiotic tools (Lorimer 2020) or alternatives to antibiotics that aim at managing microbial presence in narrower ways without threatening entire ecologies, as is the case with bacteriophages (Brives 2021). These emerging strategies contrast with antibiotic practices in the treatment of bacterial infections, which have not been especially mindful of human–microbial relations, prioritising the elimination of microbial life, often through the use of broad-spectrum medicines that target pathogenic and non-pathogenic microbes indiscriminately. Tackling AMR requires a radical move towards a more mindful enactment not just of antibiotic, but rather *biotic* relations that take seriously the challenges of multispecies coexistence and health promotion as part of more-than-human collaborations and companionship (Kirk, Pemberton, and Quick 2019). This involves understanding the role of microbes in ecosystems while acknowledging the threat some of them pose, which requires a careful, curious and attentive understanding of all elements involved.

Ambivalence towards more-than-human relations was visible in focus group discussions with breeders and veterinarians in both Benin and Burkina Faso who, when asked what was their motivation to take up livestock rearing, proclaimed to be motivated by their passion for the animals and the practice of breeding in general. They spoke of childhood affinity and their love of accompanying parents in caring for the family farm. They reflected on how business interests became more dominant as they progressed in their careers, but were clear that if one is only driven by financial gain and does not feel affection for the animals in one’s care, one will not find success as a breeder. This recognition is important because it breaks with the romantic idea that nature has intrinsic value, visibilising instead the ambivalent character of relations between humans and non-humans. Livestock mortality is a tragedy that produces negative affects if it is the result of disease, but it can have value if it occurs for the sake of food production and personal financial gain. In fact, in many situations, the value of an attachment to certain lives resides entirely in their killability, as is the case with many farm animals that are bred and nurtured—sometimes in deeply affective relations as described in the above example—because of their value as food products (Schrader *et al* 2017).

In a similar display of ambivalence, AMR and viral narratives often present microbes as having dangerous pathogenic potential (Lakoff 2015), but that can also form an important part of networks building collective resistance to disease in human and non-human communities (Beisel 2017), or even become tools for boosting immune systems (Lorimer 2016). For example, several studies have established the importance of correctly managing the microbiome of poultry litter for supporting the gut microbiota and overall health of poultry in intensive production systems (Bucher *et al* 2020; De Cesare *et al* 2019; Wang, Lilburn, and Yu 2016). Nevertheless, the authors of these studies still approached such lively matter in utilitarian terms rather than considering the affective ways in which litter management practices require attentiveness to and the careful cultivation of a beneficial litter microbiota that inhibits harmful pathogens. Breeders, on the other hand, did exhibit elements of relational thinking and regardful killing, even if they were less concerned with caring for the microbiomes of the litter or the hens. While that concern was not an explicit ethical decision in the sense we try to articulate here, it shows potential for ambivalent attitudes towards multispecies relations in the field. Indeed, the affective relations between our informants, their livestock and the environment show how the relationship between humans and their ‘natural’ surroundings are much more complex than recognising intrinsic value in non-human elements, whether these are animals or other entities that form part of the environment. Without fully recognising the complex roles that non-human actors play in the lives of humans embedded in AMR ecologies, it is hard to truly address human health needs in a manner that can also change the dynamics that currently lead to the stable increase of resistance against antibiotic practices across the globe. In other words, caring for human health needs requires caring equally for the non-human elements present in more-than-human ecologies, something that might sometimes require making ‘destructive’ decisions that are not necessarily ‘nice’ or ‘feel good’ in the sense formulated by Puig de la Bellacasa and in similar terms to those formulated by Haraway in her ethics of killing.

## Conclusion

Even if One Health has provided important steps towards recognising those elements that are not human in AMR, its implementation as a framework has so far contributed little to encourage a genuine concern for ecologies as relevant units to consider, indirectly justifying a less careful treatment of non-human health. Despite trends that indicate an increased awareness of more-than-human forms of solidarity, as the popularisation of One Health as a frame indicates, they remain far from being institutionalised, normalised or regulated (Rock and Degeling 2015). While we cannot argue that the humans involved in our examples exercise a sort of more-than-human solidarity, we can affirm that breeders do engage in relational thinking and make ambivalent decisions that involve a certain level of affective relating to their livestock. This is especially evident in care settings, like the farms we visited, but not so present in more governmental settings, like the example of the *B. microplus* dealt with by a public livestock department. More-than-human relating in locally situated settings makes evident the tension between individual and ecology in diagnostic and treatment. Ethical evaluation of such decision-making requires going beyond fixed prioritisation of the health of specific life forms to focus on the ecological, multisectoral and multispecies negotiations that take place in AMR contexts in a situated manner.

In this article, we try to support the ethical evaluation of AMR in more-than-human settings by developing a framework that takes us in an opposite direction to the anthropocentric principles that have dominated discussions of AMR ethics. The framework does this by arguing for a relational, regardful, ambivalent, caring and more-than-human approach to ecological thinking and multispecies encounters. The proposed framework uses three ethical notions developed in feminist Science and Technology studies. First, we have argued for a relational approach (Whatmore 1997) to ethics that highlights the situatedness of AMR ecologies in particular settings and economies. Second, we have argued for a care ethics approach with a more-than-human sensitivity (Puig de la Bellacasa 2017) that attends to the multispecies character of AMR and the need to frame more-than-human forms of interaction. And third, we have applied an *ethics of killing* (Haraway 2008) to advocate for a regardful attitude towards the different hierarchies that are necessarily at play in resistance multispecies encounters. Our framework is different from anthropocentric ethics in that it requires direct engagement with non-human actors that play an important role in the way humans are embedded in more-than-human ecologies. We argue that, when based on anthropocentric notions of AMR ethics, the decisions and interventions that fail to manage resistance are not ethically wrong, but at most technically inefficient (partly driven by precarity and infrastructural constraints). According to our framework, an anthropocentric perspective of those decisions is unethical not only because it fails to deal with AMR and subjugates non-human suffering to human safety, but also because such a perspective leaves us with no tools to place the priority anywhere else other than the human, even if the situation clearly requires it so.

Therefore, tackling AMR ethically from a more-than-human perspective requires more than simply addressing the health of non-human animals and the environments as a way to optimise their role in human health. AMR is defined as a One Health issue and, as such, it requires thinking in terms of the deeply relational connections that bring humans, non-human animals and the environment together. Such connections are always situated and so decision-making, research and governance require a sensitivity towards that situatedness that our framework helps to highlight. AMR research, policy and practice would benefit from shifting their thinking towards considering ecologies. Thus, we need to comprehend resistance as a form of dynamic interaction that performs response and reaction relations, something that is a constant in more-than-human ecologies. Resistance is an inevitable part of antimicrobial use and the adoption of new antibiotics will inevitably result in new forms of resistance, which means that having drug development as the only focus is an unsustainable solution to the problem of AMR. However, we can learn why and how resistance comes about as part of emerging complex relational ecologies, and modulate our practices to produce sustainable and ethical ways of acknowledging its dynamics. This is incompatible with the hierarchical approaches to AMR ethics that always submit non-human animals and the environment to humans as if they were not part of the same entanglements or as if they were mere accessories to supporting the health of human societies. Addressing AMR ethically requires attending to the agencies of other humans and non-humans present in those entanglements and acknowledging the consequences that AMR measures will have for their ways of living and dying in a more-than-human world.

## Data Availability

Data sharing is not applicable as no public data sets were generated and/or analysed for this study.

## Notes

1. In this paper, we use the notion of ecology to refer to the coexistence and co-dependency of humans, non-human animals, and the environment on microbiological forms of life, with an emphasis on the collection of practices and technologies that condition the way they interact with each other.

2. Indeed, notions of a highly interconnected world are already possible under more traditional public health frameworks dealing with zoonotic diseases that disregard the relational aspects of the different elements and actors involved in the co-emergent character of health and disease (Hinchliffe 2015).

3. Here, we choose the notion ‘more-than-human’ to describe AMR ecologies because ‘it speaks in one breath of nonhumans and other than humans such as things, objects, other animals, living beings, organisms, physical forces, spiritual entities, and humans’ (Puig de la Bellacasa 2017, 1). This can be differentiated from ‘post-human’ approaches ‘where the human is dissolved into a set of symbiotic exchanges and microbial flows’ (Lorimer 2016, 71). In a more-than-human approach the human is still a recognisable element that can emerge divergently or in consonance with non-humans.

4. Here we understand antibiotic in the wide sense of the world, as referring not only to medical products, but also to any form of governance directed at removing or eliminating microbial life (Lorimer 2020).

5. Although our informant did not clarify the specifics of this concern with the effects of antibiotics on milk, we can easily imagine some clear disadvantages since antibiotic residue in milk leads to the emergence of resistance (Sachi et al. 2019), and ignoring withdrawal periods can affect the productivity of a given batch by inhibiting dairy starter microorganisms, with the subsequent effect on cheese and fermented milk industries (Albright, Tuckey, and Woods 1961).

6. Poultry litter is the nutrient rich bedding made from organic materials enriched by the chickens’ manure.
